# Insight into the Regulation of Glycan Synthesis in *Drosophila* Chaoptin Based on Mass Spectrometry

**DOI:** 10.1371/journal.pone.0005434

**Published:** 2009-05-05

**Authors:** Yoshimi Kanie, Miki Yamamoto-Hino, Yayoi Karino, Hiroki Yokozawa, Shoko Nishihara, Ryu Ueda, Satoshi Goto, Osamu Kanie

**Affiliations:** 1 Mitsubishi Kagaku Institute of Life Sciences (MITILS), Machida, Tokyo, Japan; 2 Mitsubishi Chemical Group Science and Technology Research Center Inc., Yokohama, Japan; 3 Division of Cell Biology, Soka University, Hachioji, Tokyo, Japan; 4 Invertebrate Genetics Laboratory, National Institute of Genetics, Mishima, Shizuoka, Japan; Griffith University, Australia

## Abstract

**Background:**

A variety of *N*-glycans attached to protein are known to involve in many important biological functions. Endoplasmic reticulum (ER) and Golgi localized enzymes are responsible to this template-independent glycan synthesis resulting glycoforms at each asparagine residues. The regulation mechanism such glycan synthesis remains largely unknown.

**Methodology/Principal Findings:**

In order to investigate the relationship between glycan structure and protein conformation, we analyzed a glycoprotein of *Drosophila melanogaster*, chaoptin (Chp), which is localized in photoreceptor cells and is bound to the cell membrane via a glycosylphosphatidylinositol anchor. Detailed analysis based on mass spectrometry revealed the presence of 13 N-glycosylation sites and the composition of the glycoform at each site. The synthetic pathway of glycans was speculated from the observed glycan structures and the composition at each N-glycosylation site, where the presence of novel routes were suggested. The distribution of glycoforms on a Chp polypeptide suggested that various processing enzymes act on the exterior of Chp in the Golgi apparatus, although virtually no enzyme can gain access to the interior of the horseshoe-shaped scaffold, hence explaining the presence of longer glycans within the interior. Furthermore, analysis of Chp from a mutant (RNAi against dolichyl-phosphate α-d-mannosyltransferase), which affects *N*-glycan synthesis in the ER, revealed that truncated glycan structures were processed. As a result, the distribution of glycoforms was affected for the high-mannose-type glycans only, whereas other types of glycans remained similar to those observed in the control and wild-type.

**Conclusions/Significance:**

These results indicate that glycan processing depends largely on the backbone structure of the parent polypeptide. The information we obtained can be applied to other members of the LRR family of proteins.

## Introduction

Many proteins exposed on the surface of cells are modified with various types of oligosaccharide. For example, N-linked oligosaccharides are attached to the asparagine residues in the consensus sequence NXS/T, where X can be any amino acid other than proline. It is known that a variety of oligosaccharides are present at certain asparagine residues giving rise to different glycoforms; this consequently makes the structural and biological investigation of these molecules more difficult. In some cases, the NXS/T sequences at different sites of a single polypeptide are modified with different types of N-linked oligosaccharide, such as high-mannose, complex, and hybrid types, in a site-specific manner. However, it remains largely unknown how this site-specific modification is regulated within the Golgi apparatus.

In order to investigate these processes, we selected chaoptin (Chp), a large *Drosophila* glycoprotein containing 16 potential N-linked glycosylation sites, as a model protein [1]. Chp is required for the development and maintenance of photoreceptor cells, probably through its adhesive activity [2]–[4]. It is anchored to the extracellular surface of the plasma membrane via covalent attachment to a glycosylphosphatidylinositol (GPI) anchor [5]. On the basis of the available genetic information (Swiss-Prot/TrFMBL database: P12024), the deduced molecular mass for the 1,286 amino acids of Chp is 148,550 Da. Chp is a member of the leucine-rich repeat (LRR) (an average length of 24 amino acids) family of proteins and consists of 38 LRRs that constitute approximately 90% of the entire polypeptide. Proteins belonging to the LRR family are found in yeast, *Drosophila*, and humans [1], [6], [7], and generally fold into a non-globular horseshoe shape. Eleven-residue segments, LxxLxLxxN/CxL (where X can be any amino acid and L can also be replaced by V, I, or F), corresponding to the β-strand and adjacent loop regions are conserved in Chp and other LRR proteins [8], [9]. Thus, Chp is thought to have a folded configuration similar to that of other reported LRR family members [1], [10].

The potential N-glycosylation sites are readily identified within the Chp consensus sequence. The heavy glycosylation of Chp has been demonstrated by glycosidase treatment that dramatically reduced the apparent molecular mass of Chp (160 kDa), as estimated by sodium dodecyl sulfate-polyacrylamide gel electrophoresis. The involvement of *N*-glycans in intercellular recognition and formation of the eye structure has previously been suggested; however, the role of individual *N*-glycans at each site is not known [11]. Therefore, it is essential to reveal the structure of glycans at individual glycosylation sites, since the glycan at a particular site or a specific glycan structure might have a crucial function [12]–[16].

Despite interest in the nature of adhesion capabilities, the elucidation of glycan structure, the glycoforms, and the position of glycan attachment remains a challenging task [17]. Mass spectrometry (MS)-based methods are considered to be advantageous in this respect, owing to their high sensitivity and lower sample consumption [18]–[21]. In order to clarify the role of glycan structure in glycoproteins in general, a methodology capable of elucidating the structure of glycoproteins of a relatively large size (greater than 100 kDa) is essential. To this end, we decided to analyze the structure of Chp in order to resolve its glycan components. It is envisaged that the information thus obtained will be useful in functional investigations based on mass spectrometry.

Here, for the first time, we describe a detailed glycoform at each N-glycosylation site of Chp, an attainment achieved using a technique based on matrix-assisted laser desorption ionization time-of-flight mass spectrometry (MALDI-TOF-MS). The results revealed that high-mannose-type glycans do not co-exist with shorter glycans consisting of extra *N*-acetylgulcosamine (GlcNAc) and/or fucose (Fuc) residues at the same site, thereby giving rise to different glycoforms, and suggesting the existence of a secondary structure-dependent regulatory mechanism for glycan synthesis. To the best of our knowledge, this is the first demonstration of a relationship between protein structure and glycan processing. Having performed the Chp analysis, a knockdown experiment based on RNA interference (RNAi) against dolichyl-phosphate α-d-mannosyltransferase (Dol-P-ManTase), defects of which are related to carbohydrate-deficient glycoprotein syndrome (CDGS) type IV [22], was carried out. The knockdown experiment failed to yield any detectable eye phenotypes, suggesting that either a “sufficient amount” of wild-type equivalent Chp was synthesized and correctly expressed or that the affected part of the glycans was not essential for adhesion. However, the glycan analysis revealed similar glycan abundance at individual sites, except for the truncated glycan structure in the high-mannose-type glycoform, suggesting that glycan processing in the Golgi apparatus is restricted by protein conformation.

## Results

### Analysis Flow of Chp Glycoforms

**Figure 1:**
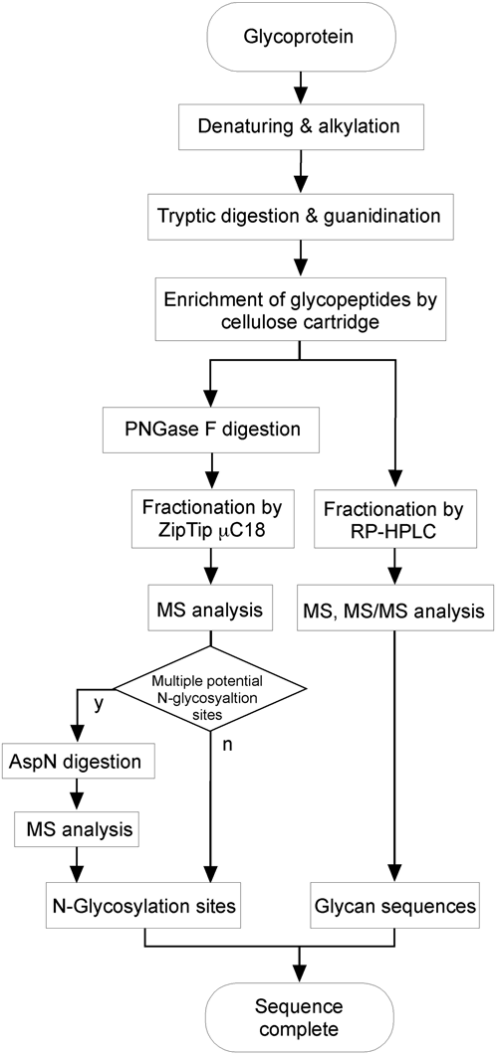
Flow chart of the sequence analysis of a glycoprotein. The purified glycoprotein is denatured and alkylated prior to tryptic digestion. The digests are subsequently subjected to guanidination in order to overcome the problem of ion suppression caused by peptides lacking arginines. Following the enrichment of glycopeptides using a cellulose cartridge column, the guanidinated tryptic digests were divided into 2 portions. One portion was subjected to PNGase F treatment followed by reverse-phase cartridge purification to determine the sites of N-glycosylation. The fraction containing multiple potential N-glycosylation sites was further treated with endoproteinase AspN. The other portion was fractionated by reverse-phase HPLC followed by MS and MS/MS analysis to obtain the glycan sequence.

The protocol followed in the current investigation is summarized in Figure 1. Briefly, Chp obtained by affinity chromatography using an anti-Chp antibody (MAb24B10) was digested by trypsin, dephosphorylated, and then guanidinated. The mixture thus obtained, which contained both peptides and glycopeptides, was passed through a cellulose cartridge column in order to enrich the glycopeptides [23]. The enriched glycopeptide fractions were used in the subsequent analyses. A portion of the crude glycopeptide was further treated with PNGase F to determine the sites of N-linked glycan attachment. This step, which involves the conversion of a glycosylated asparagine residue to aspartate, was necessary for a rapid assessment of glycan presence and location.

Mass spectrometric analysis of the tryptic digests of a glycoprotein with multiple glycosylation sites is complicated. Furthermore, the direct analysis of tryptic digests of glycoproteins is often unproductive since the presence of heterogeneity (glycoforms) at each glycosylation site means that only small amounts of individual glycopeptides are present. The analytical difficulty is compounded by the problem of ion suppression that arises from the presence of non-glycosylated peptides in a fraction. Hence, in the present investigation, the major portion of the glycopeptide mixture was further purified by HPLC and individual fractions were analyzed by MS. We decided to use the combination of a TOF mass analyzer, which is advantageous for detecting relatively large ion species, and MALDI as the ionization method, since this permits the ionization of trace amounts.

### Determination of N-Glycosylation Sites

The tryptic peptide sequences containing potential N-glycosylation sites are listed in Table 1. Twelve out of 16 potential N-linked glycosylation sites with expected *m/z* values were detected from MS analysis of the peptide fractions obtained by PNGase F treatment of enriched glycopeptides (Figure 1). From a comparison of the expected *m/z* values calculated for a peptide with known sequences and the observed values, we were able to determine the sites of *N*-glycan attachment within a margin of error.

**Table 1:** Masses of Tryptic Peptides from Chaoptin Following PNGase F Treatment.

Peptide number	Tryptic fragment	Sequence containing N-glycosylation site	Average mass of M+H^+^	Detected			
				M+H^+^ (*m/z*)	M+16+H^+^ (*m/z*)^a^	M-48+H^+^ (*m/z*)^b^	M-17+H^+^ (*m/z*)^c^
1	75–80	MV*D^77^*QSK^g^	749.83	750.4	766.4	702.7	
2	253–273	TLDISHNVIWSLSG*D^267^*ETYEIK^g^	2463.68	2462.7			
3	300–306	YFDTV*D^305^*R	915.97	916.6			
4	334–364	YC*GLT*N^339^*ISPVAFDSLVNSLQILDLSGN*D^361^*LTK^g^	3411.83	3410.9			
5	422–425	*N^422^*MTR	521.61	n.d.			
6	650–687	LEILDMAFNQLPNFNFDYFDQVGTLSNLNV*D^680^*VSHNQIR	4443.93	4442.2	4457.8	4394.6	
7	688–698	QLMY*D^692^*SSWSGR	1330.45	1330.2	1346.5	1283.2	1313.7
8	711–749	ILDLSHN*D^718^*ISIIHPGYFRPA EISLTHLHLGYNSLM*D^746^*TTR	4463.06	n.d.			
9^$^	711–728	ILDLSHN*D^718^*ISIIHPGYFR^d^	2111.40	2111.2			
10	932–946	LGLE*D^936^*V^#^SLSTVPEIR	1628.86	1627.9			
11	955–975	LGYNELPSIPQELAH*D^970^*MSNLR	2398.69	2397.6	2412.4	2349.3	
12	1000–1031	LMLSGNPITSLN*D^1012^*NSFDGVNEDLEMLDISNFR	3572.93	3571.8	3587.4	3524.4	
13	1120–1136	LT*D^1122^*ITFSGPQFTNLNER	1954.14	1955.0			
14	1143–1164	SPYLYMQLF*D^1152^*TSLQALPPNFFK^g^	2664.07	2662.6	2678.4	2616.1	
15	1171–1177	*N^1171^*ISLDIR	830.47	n.d.			

a The observed *m/z* value for the oxidized Met [M(Met)+1(H)+16(O)]^+^.

b The observed *m/z* value for the [M(Met)+1(H)+16(O)-64(CH_3_SOH)]^+^.

c The observed *m/z* value for the pyroglutamate [Q(Gln)+1(H)-17(NH_3_)]^+^.

d A peptide corresponding to a sequence P^729^–R^749^ was not observed because it was removed by the purification step using the cellulose cartridge column.

*D*: N-glycosylation site determined by PNGase F treatment.

C*: carbamidomethyl cysteine.

K^g^: guanidinated lysine.

V^#^: undetermined.

Underline: non-glycosylated *N*.

n.d.: not detected.

The sequence of 9^$^ is a fragment of 8.

Peptide modifications are often observed in mass spectra and provide useful information for the annotation of amino acid sequences. Methionines in tryptic peptides (see Nos. 1, 6, 7, 11, and 14 in Table 1) were oxidized, and thus ions such as [M(Met)+1(H)+16(O)]^+^ and [M(Met)+1(H)+16(O)−64(CH_3_SOH)]^+^ were observed as well as a protonated molecule, [M(Met)+1(H)]^+^. Further, glutamine at the N terminus (see No. 7 in Table 1) was partially observed as a pyroglutamate [Q(Gln)+1(H)−17(NH_3_)]^+^ [24] (Figure S1). A sequence for the peptide I^711^–R^728^ (No. 9) was obtained even though there was no report that trypsin cleaves the linkage between arginine and proline for a sequence of …FRP…

Among the peptides that were confirmed to be N-glycosylated, peptide Y^334^–K^364^ (No. 4) had 2 potential N-glycosylation sites (N^339^ and N^361^). It appeared from the MS analysis of N-glycosylation sites that an increase in *m/z* value by only 1 amu could not be reliably made for the analysis of ions over *m/z* 3000. As an alternative, we decided to treat the peptides obtained by PNGase F treatment with AspN endoproteinase. AspN cleaves the N terminus of aspartate (D), and therefore should produce a new peptide terminating with D instead of N. As a result, the peptide YCGLT*N* ^339^ISPVAFDSLVNSLQILDLSGN*N* ^361^LTK yielded D^361^LTK and YCGLTN^339^ISPVAF in which only N^361^ was converted to D, indicating that this particular position in the peptide was N-glycosylated (Figure S2).

With regard to positions N^422^ and N^1171^, it was not possible to ascertain the presence or absence of a glycan since glycanase is unable to hydrolyze glycans when the N-terminal asparagine is glycosylated [25]. Therefore, we performed MS/MS analysis of the RP-HPLC fractions (Figure 1) in order to confirm the presence of a glycan at these sites. One of the asparagine residues (N^1171^) that was not initially identified as being glycosylated was additionally established to be a site carrying an *N*-glycan based on MS/MS analysis of the glycopeptide fraction. Thus, a total of 13 *N*-glycosylation sites were confirmed among the 16 potential sites.

### Glycoform Analysis at Each Glycosylation Site

**Figure 2:**
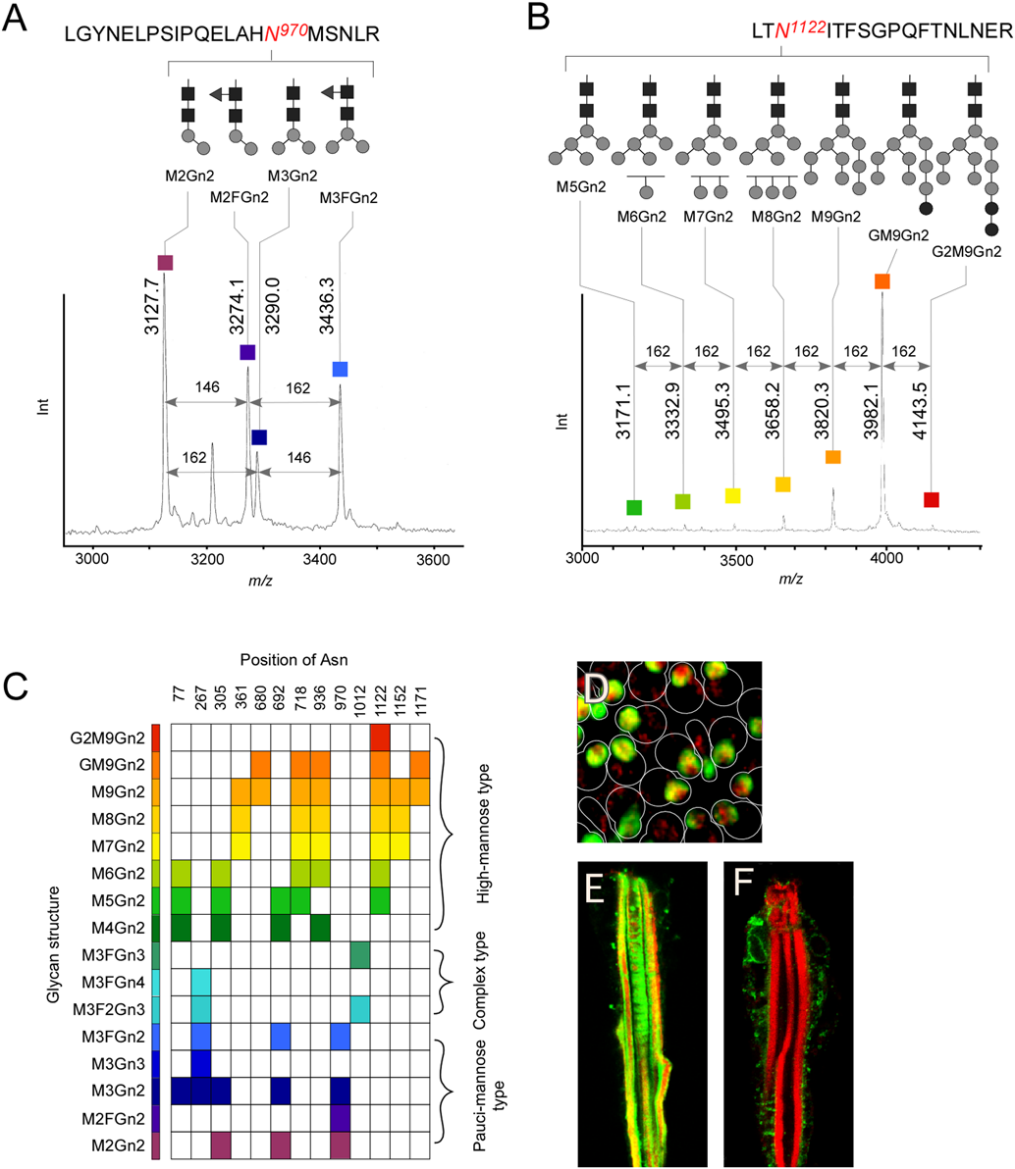
Mass spectra and structural variation of the *N*-glycans of chaoptin. (A) Mass spectrum of a glycopeptide fraction containing pauci-mannose-type glycans following RP-HPLC. The structures of the 4 glycans observed at N^970^ are shown schematically. The glycopeptide signals are spaced by 162 amu (mannose, M) or 146 amu (fucose, F). (B) Mass spectrum of a glycoprotein fraction that contains high-mannose-type glycans in which 7 glycans are observed at N^1122^. The signals spaced by 162 amu correspond to a number of mannose (M) or glucose (G) residues. (C) Observed glycan structures at each glycosylation site are shown. The horizontal and longitudinal axes show the position of asparagine (Asn) in the polypeptide and glycan structures, respectively. Distinctive colors are assigned to the individual glycan structures, which are used in the paper hereafter. (D) The majority of Chp is localized in the rhabdomeres as determined by staining with anti-Chp antibody and Alexa Fluor 488-conjugated anti-mouse IgM. TMR (tetramethylrhodamine)-phalloidin staining against F-actin. (E) A longitudinal conforcal image: same staining as in D. (F) Photorecepter cells were stained (longitudinal image) using TMR-phalloidin and anti-KDEL antibody and Alexa-Fluor-488 conjugated anti-mouse IgG.

Following separation by reverse-phase (RP) HPLC, the glycopeptide fractions were analyzed by MALDI-TOF-MS and MS/MS. The fractions containing glycopeptides were identified in a chromatogram by the presence of a series of signals due to the glycoform. The retention of glycopeptides depends on the hydrophobicity associated with hydrophobic residues in peptides; thus, a series of glycopeptides sharing the same peptide sequence results in a series of peaks. Following MS, the glycopeptide peaks were shown to differ by *m/z* 203 (GlcNAc, Gn), 162 [mannose (Man), M or glucose (Glc), G], or 146 (Fuc, F). We assessed glycan composition on the basis of the *m/z* values only because it is practically impossible to determine the linkage position and anomeric configuration based on the current setup of the MS experiment without methylation that provides information regarding linkage position. Examples of the mass spectra of glycopeptides with different glycoforms are shown in Figure 2A and 2B. Analysis of the glycopeptides containing N^970^ revealed the presence of 4 glycans that were of the pauci-mannose type (Figure 2A). The glycoform found at N^1122^ consisted of high-mannose-type glycans containing 5 to 11 hexoses (Figure 2B). We were concerned that immature glycoproteins being synthesized in the endoplasmic reticulum (ER) and Golgi might be analyzed together with mature forms. However, immunostaining of photoreceptor cells using anti-Chp antibody (24B10) revealed that the Chp was accumulated in the rhabdomeres, indicating that the majority of the glycoproteins we analyzed were of the mature form (Figure 2D–2F).

*Drosophila N*-glycans are classified into 3 types; namely, high-mannose, pauci-mannose, and complex types. The relationship between observed glycan structure and the site of attachment is shown in Figure 2C, where the numbers along the horizontal axis indicate the position of the observed N-glycosylated asparagine in the polypeptide sequence, and the color indicates observed glycan structures. High-mannose-type glycans were observed at 10 positions (N^77^, N^305^, N^361^, N^680^, N^692^, N^718^, N^936^, N^1122^, N^1152^, and N^1171^). In contrast, complex-type glycans were observed at 2 positions (N^267^ and N^1012^), whereas pauci-mannose-type glycans were observed at 5 positions (N^77^, N^267^, N^305^, N^692^, and N^970^). The glycans at positions N^77^ and N^305^ contained relatively short high-mannose-type and pauci-mannose-type glycans consisting of only Man residues other than chitobiose, whereas the glycans at position N^692^ contained a Fuc residue. The distribution of glycoforms at each site appears to be controlled since high-mannose-type glycans and shorter glycans that contain extra GlcNAc and/or Fuc residues (pauci-mannose and complex types) do not co-exist at the same site, thereby giving rise to different glycoforms. On the basis of these observations, it would appear that the state of the glycoform is, to a certain extent, dependent upon its position within the peptide.

**Figure 3:**
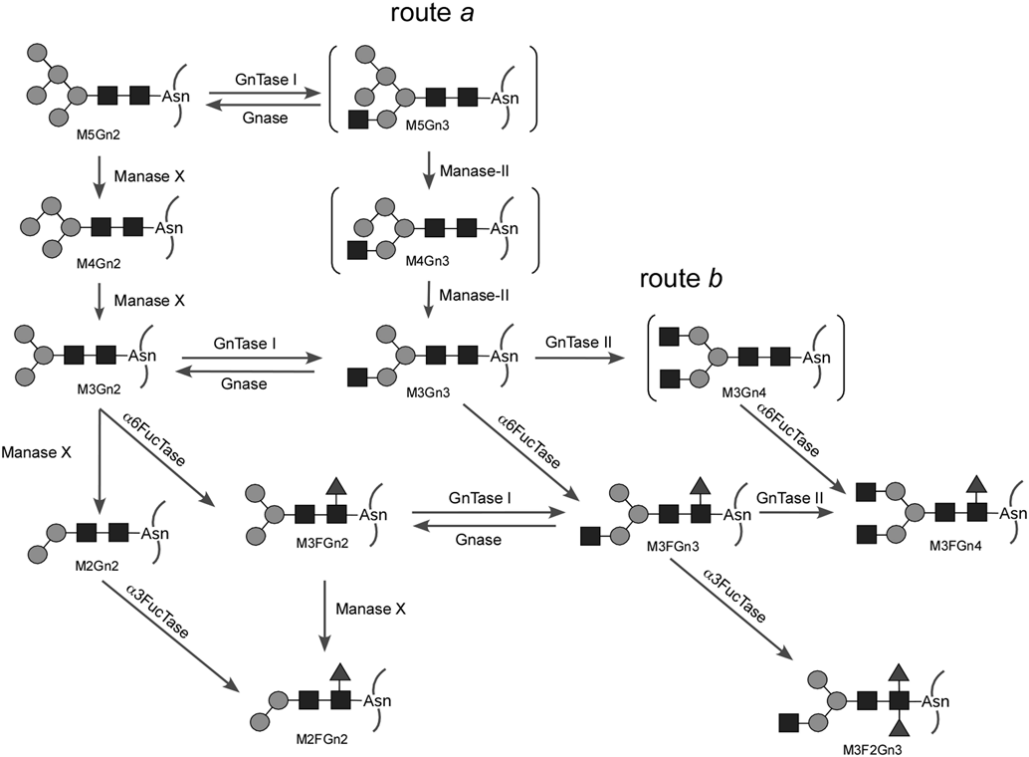
Schematic representation of *N*-glycan processing in *Drosophila*. The *N*-glycan synthetic pathway in wild-type *Drosophila* is shown. The scheme was created based on a previously reported synthetic pathway and the results presented in Figure 2C. An enzyme indicated as Manase X has not been identified. Route *a* and route *b* are the predicted pathways, although intermediates, described in parentheses, within the 2 pathways have not been observed.

The glycan structures expected from the observed glycan compositions are summarized in the predicted synthetic pathway depicted in Figure 3. This confirmed the presence of most of the reported glycan structures, although some of the important intermediates are absent. The structures indicated in parentheses in route *a* (M5Gn2→M5Gn3→M4Gn3→M3Gn3) and route *b* (M3Gn3→M3Gn4→M3FGn4), which are the accepted synthetic pathways, denote undetected glycans; these structures could exist as intermediates, although they may be present in undetectable amounts (see Discussion).

Glycan structures can be categorized by the processing enzymes involved in their synthesis. GM9Gn2 (Figure S3A), which is processed by α-glucosidase I and II (Glcase I and II) following attachment of the oligosaccharide G3M9Gn2 to a polypeptide in the ER, but not completely processed by Glcase II in the ER, was found at positions N^680^, N^936^, N^1122^, and N^1171^. Glycan structures formed by the α-mannosidase I (Manase I) reaction in the Golgi apparatus, subsequent to the reactions of Glcase I and II in the ER, were observed at positions N^361^ and N^1152^. High-mannose-type glycans were also observed at positions N^77^, N^305^, and N^718^. The glycans at N^77^ and N^305^ were of a pauci-mannose type, although the glycan at N^718^ was of a high-mannose type only. These glycans are thought to be the products formed by the reactions of Manase without any subsequent reactions mediated by *N*-acetylglucosaminyltransferase I (GnTase I) and fucosyltransferase (FucTase).

In contrast, the products of the sequential reactions of both GnTase I and FucTase(s), which are classified as the complex type, were observed at positions N^267^ and N^1012^. These glycans contained both α-(1→3)- and α-(1→6)-linked Fuc residues. It has been reported that α-(1→3)-linked Fuc is abundant in the neural system [26], an observation that has been confirmed by using anti-HRP antibody [27]. Although we confirmed the presence of the α-(1→3)-linked Fuc, immunostaining experiments using anti-HRP antibody indicated that the residue was not abundant in Chp [28]. In *Drosophila*, Fuc can be attached to the core GlcNAc by α-(1→3) and/or (1→6) linkages [29]. Thus, the linkage position of mono-fucosylated glycans, which are of the pauci-mannose type, in the glycoforms at N^267^, N^692^, N^970^, and N^1012^ could not be determined by MS (Figure S3B). The knockdown mutant analysis of α-(1→3) FucTase or α-(1→6) FucTase, or more practically PNGase A treatment [30], could provide more detail information, although we may be able to determine glycan structure by referring to the synthetic pathway [31]–[33].

### Relationship between Protein Conformation and Glycan Structure

**Figure 4:**
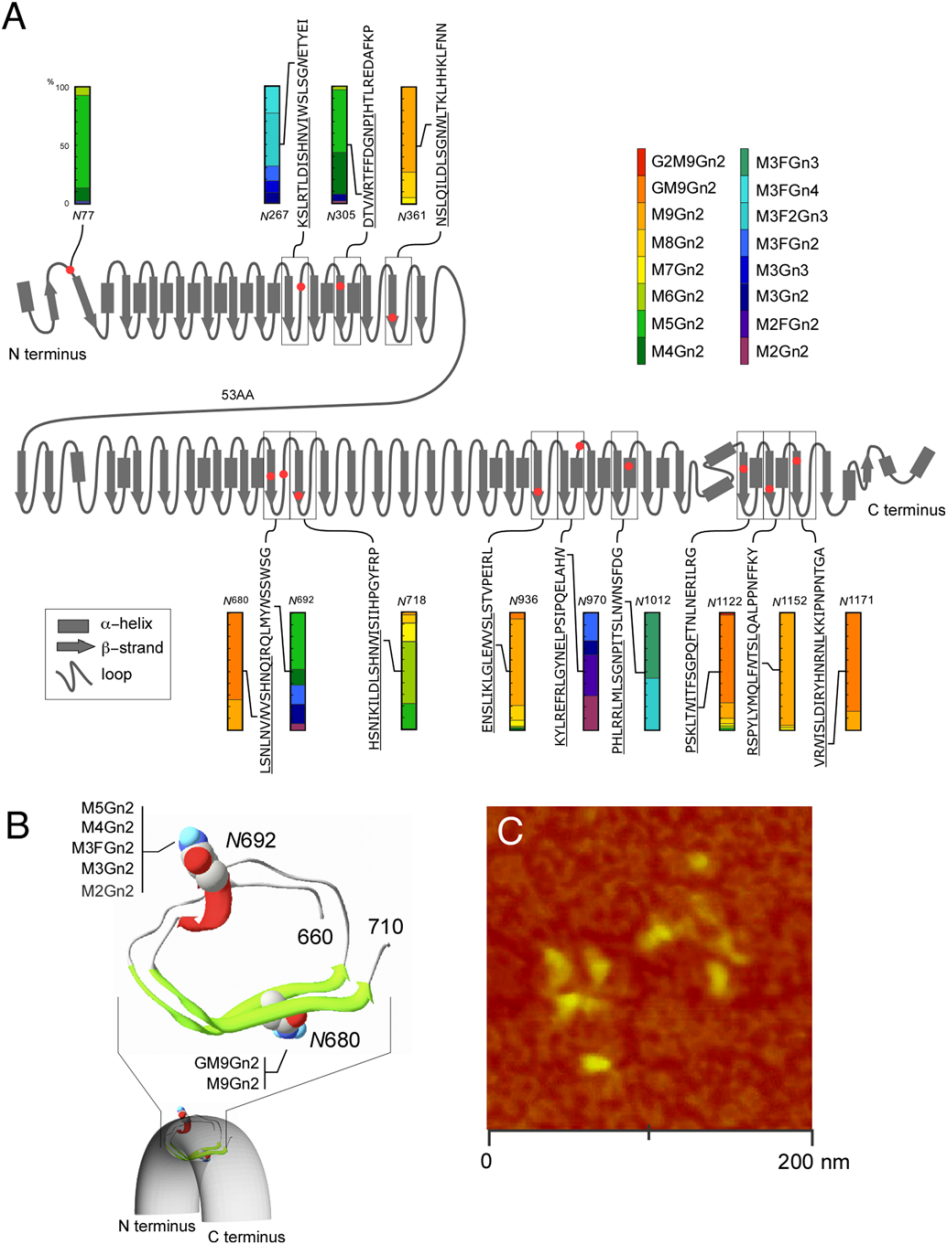
Predicted secondary structure of chp with details of the glycoforms at each glycosylation site. (A) Protein secondary structure was predicted by PSIPRED ver. 2.6. N-glycosylation sites are indicated by red dots and the contents of individual glycoforms are shown (color coordinated with those in Figure 2). (B) A slice section of LLR (AA^648^–AA^729^) with β-strands located on the inner side and α-helices or coils located on the outer side. The positions of N-glycosylation sites, N^680^ carrying high-mannose-type glycans only, and N^692^ carrying shorter glycans are shown as space-filled representations. The model was generated using Swiss-PdbViewer ver. OSX 3.9b2. (C) Atomic force microscopy image of Chp (wild-type with glycans attached) showing the overall horseshoe-shaped structure. The image shows possibly 8 individual Chp molecules. Each object represents a single Chp molecule carrying one of the glycoforms. Most of the molecules have similar arch-shaped structures with some variations. This might reflect an inherent feature of the glycoform but is considered more likely to be due to the resolution of the method used.

In Figure 4, we present a schematic representation of Chp in order to illustrate the relationship between glycan structure and protein conformation based on the characteristic folding of the LRR motif. Figure 4A shows the secondary structure of Chp predicted by PSIPRED ver. 2.6 (http://bioinf.cs.ucl.ac.uk/psipred/psiform.html) in which the glycan attachment sites and glycoform contents are indicated. The polypeptide structure of Chp is thought to be constructed of a β-strand and α-helix or to be alternately coiled owing to the LRR motifs (Figure 4B). Although the crystal structure of Chp is not currently available, the conformation might be similar to that of other LRR family proteins, e.g., the Toll-like receptor [34]. Furthermore, this conformation was confirmed by the observation of arch-shaped Chp molecules by atomic force microscopy (Figure 4C). The fact that the individual molecules observed had an arch shape suggests that the Chp folded correctly overall. This was also supported by the localization of the glycoprotein in the rhabdomeres.

In Figure 4A, the content of color-coordinated glycan structures are indicated as percentages estimated from the height of the signals in the mass spectra for individual sites (also see Figure 2C). This representation was made possible based on the fact that the ionization tendency of glycopeptides depends mostly on the peptide portion; thus, quasi-quantitative data analysis of a glycopeptide can be performed [35], [36]. The spectrum shown in Figure 4A clearly indicates that high-mannose-, pauci-mannose-, and complex-type glycans exist independently at different positions within the Chp polypeptide.

With the exception of N^77^, which is not located in the LRR, the N-glycosylation sites attached with high-mannose-type glycans are located in the β-strand region in the interior of the arch. At other N-glycosylation sites located outside of the β-strand, the glycans at positions N^267^ and N^1012^ were found to be of a complex type. Moreover, the glycans at positions N^692^ and N^970^ were found to be of a pauci-mannose-type. These glycans are thought to be located in either the loop or the α-helix at the exterior of the arch-shaped Chp. These results revealed that complex- and pauci-mannose-type glycans are located on the more exposed surface of the protein.

### Glycan Analysis of Chp from a Knockdown Mutant

The results of the glycoform analysis described above reveal not only the structures of the glycans but also information on the enzymes involved in their synthesis. Consequently, an analysis of the relevant mutants could provide important insights into the regulation of glycan synthesis. In order to confirm the quasi-quantitative analysis of a glycoform based on the MS of mutants and to obtain control values for analysis of the subsequent knockdown experiments prior to interference, we initially performed an analysis of organisms carrying green fluorescent protein-inverted repeat (GFP-IR) as a control [37].

**Figure 5:**
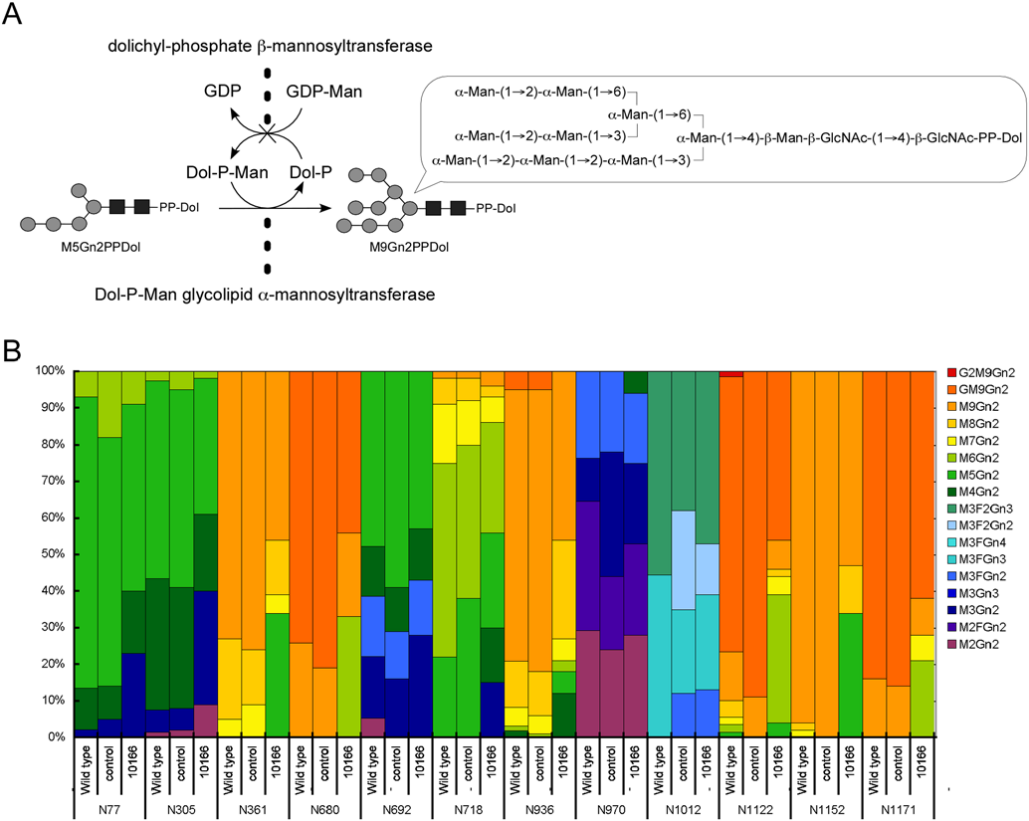
M9Gn2PPDol synthesis and the relative abundance of *N*-glycan in wild-type, control, and mutant (*CG10166*) *Drosophila*. (A) Dolichyl-phosphate α-d-mannosyltransferase (Dol-P-ManTase) [2.4.1.83], is converted to Dol-P-Man from GDP-Man, and serves as a donor substrate in the synthesis of M9Gn2PPDol from the donor M5Gn2PPDol in *N*-glycan synthesis. (B) Contents of glycoforms at individual glycosylation sites are indicated as percentages (colors are coordinated with those in Figure 2 and Figure 4). Due to the detection limit of mass spectrometry, the glycopeptide signals at position N^267^ in the mutant did not provide sufficient intensities to enable an estimation of glycoform abundance; thus, the data are not shown. Observed glycans are M3, M3F, GnM3F, and Gn2M3F.

In addition, we analyzed Chp from an organism in which the gene (*CG10166*) encoding the enzyme Dol-P-ManTase [2.4.1.83] had been knocked down (Figure 5). Deficiency in this gene is known to cause CDGS type IV in human. Patients with this condition are characterized by the production of a truncated lipid-linked oligosaccharide containing only 5 Man residues, whereas normal individuals produce an oligosaccharide containing 9 Man residues (Figure 5A). Although this oligosaccharide is faithfully transferred to a protein, subsequent oligosaccharide processing is abnormal in the hybrid-type glycans [22]. Dol-P-ManTase transfers a Man residue from GDP-Man to dolichyl-phosphate, and the resulting product, dolichyl-phosphate α-d-mannose (Dol-P-Man), is used as a substrate in the synthesis of M9Gn2PPDol from M5Gn2PPDol (Figure 5A). Dol-P-Man is also used as a substrate in the synthesis of the GPI anchor. Thus, the knockdown of Dol-P-ManTase may disrupt the synthesis of both *N*-glycan and the GPI anchor owing to the resulting shortage of Dol-P-Man. Since Chp is a glycoprotein linked to a GPI anchor, the correct expression of Chp at the plasma membrane may rely on the complete synthesis of the GPI anchor as well as the *N*-glycans. An additional important objective in this context is to provide evidence for the suggested regulation of glycan processing that relates to the conformation of the Chp polypeptide. The knockdown of Dol-P-ManTase would result in a shortage of Dol-P-Man, and thus yield Chp with truncated glycans.

The differences in glycan structure can also be addressed by considering processing on the interior and exterior of the arched-shape Chp molecule. First, the overall profile of the observed glycoform of the control and the wild-type closely resembled each other showing good reproducibility where the MS-based analysis could be performed in a quasi-quantitative manner (Figure 5B). Analysis of Chp from the *CG10166* mutant revealed that it exhibited no obvious physical phenotypes. Furthermore, detailed analysis of the mutant revealed that the constitution of the rhabdomeres was not affected, as confirmed immunohistochemically using an anti-Chp antibody. Moreover, an accumulation of Chp in the ER was not evident from these experiments (Data not shown). Thus, it was considered that a sufficient amount of Dol-P-Man was synthesized under the RNAi conditions. On the basis of the glycoform analysis of Chp in the *CG10166* mutant, it was determined that the structure of only the high-mannose-type glycans was affected, whereas the distribution of pauci-mannose- and complex-type glycans remained similar to those observed in the control and wild-type. It is conceivable that M5Gn2PPDol was transferred to Chp polypeptide instead of M9Gn2PPDol in patients with CDGS type IV. Furthermore, the shorter glycans transferred to the outer surface of Chp were correctly processed, which suggests that the oligosaccharyltransferase, Glcases, Manase I, and possibly GnTase I do not require the missing branch structure for substrate recognition. In spite of these features, glycans containing shorter chains attached to the β-strands were not further processed, which strongly suggests the involvement of steric factors in the regulation of glycan processing.

**Figure 6:**
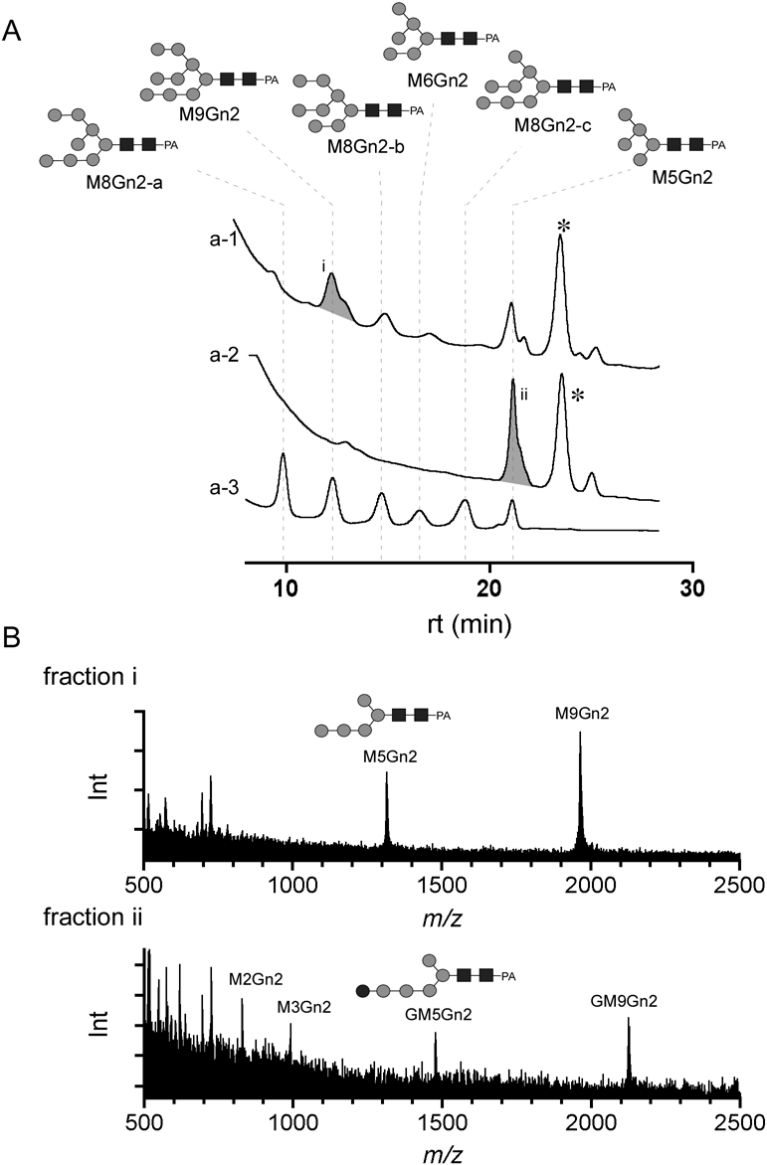
Mutant Glycan analysis based on HPLC and mass spectrometry. (A) RP-HPLC chromatograms of pyridylaminated (PA)-sugars, which were prepared from mutant glycopeptides containing the following N-glycosylation sites: N^936^ and N^1152^ (a-1), and N^680^, and N^1122^ (a-2). Chromatogram a-3 shows commercially available PA-sugars as a reference. A peak of unknown identity indicated by an asterisk that might be associated with a reagent used for the fluorescent labeling appeared in all fractions. (B) Mass spectra of peak i (a-1) and peak ii (a-2) from the chromatograms shown in (A). M9Gn2 and M5Gn2 were observed in the fraction containing peak i. GM9Gn2 and GM5Gn2 were observed in the fraction containing peak ii.

Since a portion of the transferred glycan structures was shorter than normal in circumstances where glycan processing was affected by the RNAi, we performed a further structural analysis of these glycans. The HPLC fractions of glycopeptides containing M9Gn2 or GM9Gn2 were treated with PNGase F in order to release glycans; these glycans were then pyridylaminated and separated by RP-HPLC (Figure 6A). The fractions thus obtained were analyzed by MS. This analysis revealed the presence of the glycans M5Gn2 and GM5Gn2 that lack an entire branch on α-Man-(1→6)-β-Man in fractions (i) and (ii), respectively (Figure 6B).

## Discussion

Most secreted and membrane-anchored proteins are posttranslationally modified. One of the major types of protein modification is glycosylation; however, the regulation of this process is not fully understood. The difficulty in characterizing this process lies in its template-independent nature in the Golgi apparatus. The investigation of this mechanism is of importance since it is well known that changes in the structures of glycans attached to a protein occur depending on the status of a cell and that such changes are also related to certain diseases [22]. We investigated the relationship between glycan structure and backbone protein folding using Chp, a glycoprotein found in *Drosophila* photoreceptor cells, which has 16 potential *N*-glycan attachment sites. The protein is a member of the LRR family of proteins and is thus thought to fold into an overall horseshoe-shaped structure. Our detailed analysis of the site-specific *N*-glycan structures based on MS confirmed the presence of 13 N-glycosylation sites, and, for the first time, identified the individual glycoforms at each site. These results highlight 2 important aspects of glycan processing: (1) the nature of the synthetic pathways, since glycans are synthesized by the sequential action of multiple enzymes; and (2) the relationship between glycan structure and protein conformation based on the characteristic folding of LRR family proteins.

Although knowledge of the synthetic pathways of *N*-glycans in *Drosophila* has been limited to the difference between hybrid-type and pauci-mannose-type glycans [38], the synthetic pathways could be predicted in detail based on the observed glycan structures (Figure 3). We first focused on the synthetic pathway of pauci-mannose-type glycans. It is conceivable that there are 2 routes to produce M3Gn3. Manase II is known to act on substrates after the transfer of Gn onto M5Gn2 (route a) [39], [40]; however, intermediates such as M5Gn3 and M4Gn3 (shown in parentheses in Figure 3) were not detected in our analysis. In contrast, M4Gn2 and M3Gn2, which lack certain Man residues without additional Gn, were observed. This does not necessarily preclude the presence of route *a*, but it does suggest the following 2 possibilities: (1) the addition of a Gn residue may not be required for the Man cleavage in *Drosophila*, and (2) the reaction rate of GnTase I might be considerably faster than that of Manase II. A study of Manase II-deficient mice suggested that an alternative enzyme with overlapping activity might be responsible for the processes yielding the observed structures (shown as Manase X in Figure 3) [41]. *Drosophila* might have other Manases, or one having a substrate specificity different to that of mammalian Manase I. In this respect, a glycoform analysis of a Manase mutant could provide further information. Another interesting observation was that no intermediates of the structure M3Gn4 (indicated with parentheses in Figure 3) were found in the expected pathway (route b). This also suggests the presence of an alternative route.

Furthermore, the existence of different types of glycans at different positions in the Chp molecule makes it possible to discuss the relationship between the regulation of glycan structure within the protein conformation and the reactions of glycan-processing enzymes. Among the different types of glycans, those mainly containing M9Gn2 and GM9Gn2 were found in the β-strand regions. We cannot exclude the possibility that the observed G2M9Gn2 (N^1122^) and GM9Gn2 (N^680^, N^718^, N^936^, N^1122^, and N^1171^) associate with immature glycoprotein in the ER (Figure 2C); however, localization of Chp clearly revealed the accumulation of Chp in the rhabdomeres (Figure 2D–2F). Taking the observed intensities of GM9Gn2 (Figure 2B) into account, we believe that the majority of glycoproteins we analyzed were mature. It is suggested that the bulky glycans are located in the interior of the Chp arch (Figure 4A–4C). On the other hand, it was found that complex-type and pauci-mannose-type glycans were located on the more exposed surface of the protein. Various processing enzymes act on the exterior of Chp, although virtually no enzyme can gain access to the interior of the horseshoe-shaped scaffold, hence explaining the presence of longer glycans within the interior. These observations were further corroborated by a knockdown experiment. Mutant analysis revealed that truncated *N*-glycan structures were processed in the same manner. The changes in *N*-glycan structure occurring in the ER did not affect further modification of the Chp glycans in the Golgi apparatus, and the results strongly suggested the presence of a regulation of *N*-glycan processing based on the 3-dimensional structure of the backbone polypeptide [12], [13].

Information regarding the quality control was also obtained from mutant analysis. The protein sorting of Chp was normal without any accumulation in the ER. This suggests that the dolichyldiphosphooligosaccharide-protein oligosaccharyltransferase did not require modification (chain elongation) onto α-Man-(1→6)-β-Man. Although it was shown that the oligosaccharyltransferase recognized the shorter glycan as a substrate, the transfer efficiency might be affected. This was evident from the HPLC analysis of glycopeptides released from the control and mutant Chp, which revealed a decrease in the total amount of glycopeptide in the mutant (Figure S4). This observation is well supported by the observation of an increase in the incompletely glycosylated transferrin in a patient with CDGS type IV [22].

In addition, the abovementioned structures were not important for the quality control by Calnexin (CNX) and/or Calreticulin (CRT) [42], [43]. Indeed, it is also conceivable that Chp might not require these controls for correct folding since the normal distribution of Chp was observed in a cnx99A knockdown fly [44]. It should be noted that, owing to the nature of the knockdown experiments, normal glycans were also found; however, the detection of glycans carrying Glc residue(s) (GM9Gn2), such as those at positions N^680^, N^936^, N^1122^, and N^1171^, indicates that modification of at least these locations was not important for the quality control of Chp [45].

To conclude, our analysis of one of the major glycoproteins in *Drosophila* photoreceptor cells, chaoptin (Chp), confirmed the presence 13 N-glycosylation sites out of 16 potential sites. On the basis of the MS and MS/MS analyses of proteolytic digests, it is suggested that *N*-glycan processing in Chp depends largely on protein folding. Various processing enzymes act on the exterior of Chp, although virtually no enzyme can gain access to the interior of the horseshoe-shaped scaffold, hence explaining the presence of longer *N*-glycans within the interior. These observations were further corroborated by a knockdown experiment. Although the crystal structures of some LRR proteins have recently been determined [34], no detailed information is currently available regarding glycans since these are cleaved prior to crystallization. The information we obtained can be applied to other members of the LRR family of proteins. We also demonstrated, firstly, that quasi-quantitative analysis was possible by performing analyses of both wild-type and mutant Chp glycans, and, secondly, that immature glycan structures lacking α-Man-(1→2)-α-Man-(1→3) and α-Man-(1→2)-α-Man-(1→6) disaccharides, which under normal circumstances should be attached to an α-Man-(1→6)-β-Man branch, did not affect glycan maturation, and that the glycan structure might not be important for the correct folding based on CNX and/or CRT in Chp. On the basis of the observed glycan structures, alternative glycan processing routes can be predicted where these structures suggest the presence of a new route [41]. Finally, it should be noted that the MS-based technique described herein could also be applied to the investigation of other glycoproteins, since it is still quite difficult to perform analysis of large glycoproteins with molecular masses exceeding 100,000 Da. It is important to establish such a method as an analytical tool for assessing any changes in glycan structures related to disease state, differentiation, cell types, and glycoprotein as pharmaceutical.

## Materials and Methods

### Preparation of Chaoptin from *Drosophila* Heads

Heads of adult wild-type Canton-S *Drosophila melanogaster* were homogenized in lysis buffer consisting of 10 mM sodium phosphate (pH 7.0), 100 mM NaCl, 0.5% Triton X-100, and complete protease inhibitor (Roche), using a Teflon homogenizer. The lysate was centrifuged at 13,000×*g* for 10 min in order to remove nuclei and debris. Clarification of the lysate was achieved by passing it through a 0.22-µm PES filter (Millipore). Immunoaffinity purification of Chp was performed using columns of protein G-Sepharose (GE Healthcare), to which anti-Chp antibody (24B10) (University of Iowa DSHB) had been covalently coupled [46]–[48]. The lysate was incubated with 24B10/protein G-Sepharose for 2 h. The affinity column was subsequently washed with the lysis buffer minus protease inhibitor, followed by 50 mM triethylamine-acetic acid (pH 4.5). Chp was eluted with 50 mM triethylamine-acetic acid (pH 3.5).

Mutant fly–RNAi fly lines were generated as described previously [37]. Two transgenic fly stocks, GAL4-driver and upstream activating sequence system-inverted repeat (UAS-IR), were used in this system. The GAL4-driver fly has a transgene containing the yeast transcriptional factor GAL4, the expression of which is under the control of a cytoplasmic actin promoter. The UAS-IR fly has a transgene containing the inverted repeat of the target gene ligated to the UAS promoter, a target of GAL4. In this investigation, GMR-GAL4 was used to eye-specifically control RNA expression. In the F1 progeny of these flies, the double-stranded RNA of the target gene is expressed ubiquitously in all cells to induce the gene silencing.

### Preparation of Glycopeptides

RapiGest (Waters) and dithiothreitol were added to 200 µl of a 0.1 µg/µL protein solution (adjusted to pH 8.0 with 1 M ammonium bicarbonate) to final concentrations of 0.1% (w/v) and 5 mM, respectively. This mixture was then incubated for 30 min at 60°C. Iodoacetoamide was added to a final concentration of 15 mM, and reacted in the dark at room temperature for 30 min. Modified trypsin (Promega) was then added to a final trypsin/glycoprotein ratio of 1/50 (w/w) and CaCl_2_ solution was added to a final concentration of 1 mM. Tryptic digestion was achieved by incubating overnight at 37°C. The digestion was terminated by the addition of 1% trifluoroacetic acid (TFA). In order to remove salt and trypsin, the tryptic digest was applied to a Sep-Pak Light C18 cartridge column (Waters). The peptide mixture was eluted using 60% acetonitrile containing 0.1% TFA (4 mL) followed by washing with 0.1% TFA (10 mL). Dephosphorylation was achieved by incubating at 37°C overnight with alkaline phosphatase (2.4 U; Toyobo) in 200 µL of 50 mM ammonium bicarbonate (pH 8.0) containing 10 mM MgCl_2_. The peptide mixture was guanidinated using a Guanidination kit (SIGMA) following the manufacturer's protocol [38], [49]. The reaction mixture was dried and dissolved in 500 µL of 500 mM NH_4_OH. This resuspension was then mixed with 250 µL of 500 mM *O*-methylisourea hemisulfate. Following incubation of the mixture at 60°C for 30 min, 750 µL of 10% TFA was added. A quarter of the reaction mixture was applied to a cellulose cartridge column, and the peptide mixture was eluted with 3 mL ethanol/75 mM ammonium bicarbonate (1∶2) followed by washing with 10 mL *n*-butanol/ethanol/water/acetic acid (4∶1∶0.97∶0.03).

### Peptide:*N*-glycanase F (PNGase F) Digestion of Glycopeptides

A half volume of the cellulose cartridge eluent was dried and dissolved in 50 µL of 20 mM phosphate buffer (pH 7.0). PNGase F (0.5 U) was added to the peptide solution and digestion was achieved by incubating at 37°C overnight. A portion of the peptide mixture was desalted using a ZipTip μ-C18 prior to MS analysis.

### HPLC Separation of Glycopeptides and Peptides

Glycopeptide and peptide mixtures were separated by RP-HPLC using a 1.0-mm i.d.×100 mm, 5-µm Inertsil C18 column (GL Science). All separations were performed using a mobile phase of 0.1% TFA and 70% acetonitril containing 0.1% TFA with a linear gradient mode at 35°C. The flow rate was set at 50 µL/min and UV detection was performed at 215 nm.

### Endoproteinase Asp-N Digestion of Peptides

HPLC peptide fractions were dried and dissolved in 20 µL of 20 mM phosphate buffer (pH 7.0). To each mixture, 0.5 units of endoproteinase Asp-N were added. The digestion was carried out at 37°C for 3 h. The mixture was desalted using a ZipTip μ-C18 prior to MS analysis.

### MS Analysis of Glycopeptides and Peptides

The matrix for peptide analysis by MALDI-TOF-MS consisted of either 10 mg/mL cyano-4-hydroxycinnamic acid in 50% acetonitril or 0.1% TFA or 10 mg/mL 2,5-dihydroxybenzoic acid in 10% ethanol. MALDI-TOF-MS spectra were acquired using a Voyager mass spectrometer (Applied Biosystems, Foster City, CA). Mass spectra were acquired in the positive linear mode. MS/MS measurements were performed using an Ultraflex TOF/TOF mass spectrometer with the LIFT-MS/MS facility in the positive mode. (Bruker Daltonics GmbH, Hamburg, Germany).

### Phalloidin- and Immuno-histochemistry

Adult Canton-S *Drosophila* were anesthetized with CO_2_ and then decapitated. Retinas were excised from the heads using a razor blade and a tungsten needle, immersed in 4% paraformaldehyde in PBS containing 0.1% Triton X-100, and then incubated on ice for 1 h. After rinsing in 0.1% Triton X-100 in PBS, the specimens were incubated in 0.1% Triton X-100 and 0.1% BSA in PBS (PBSTB) for 30 min at room temperature. The specimens were then incubated with anti-Chp antibody (24B10) in PBSTB for 2 h at room temperature. Following a rinse in PBSTB, the specimens were incubated with rhodamine phalloidin (1∶20; Invitrogen) in PBSTB for 2 h at room temperature. The secondary antibody used was Alexa Fluor 488-conjugated anti-mouse IgG (1∶200; Invitrogen). After rinses in PBSTB, the specimens were mounted with Vectashield (Vector Laboratories, Burlingame, CA, USA) and observed using a FluoView FV500 Laser Scanning Confocal Microscope (Olympus, Tokyo, Japan).

### Atomic Force Microscopy (AFM)

Freshly cleaved mica surfaces (20×20 mm; Ted Pella, Redding, CA, USA) were incubated with 20 µL 0.01% aminopropyltriethoxysilane (Gelest Inc.) water solution at room temperature for 10 min, which was subsequently aspirated off. A 5-µL drop of cosmid DNA 380H5 (0.1 µg/mL) bearing elongation complexes was then spread on a mica surface by covering it with a clean glass coverslip. The coverslip was peeled off after 5 min, and the mica surface air-dried before imaging. Using a BioScope system with a NanoScope IIIa controller (Digital Instruments, Santa Barbara, CA, USA), tapping mode scans on mica in air were performed with Metrology Probe Tap 300 tips (Ted Pella).

### Pyridylamination of Glycans

HPLC fractions of glycopeptides were treated with PNGase F and then dried. The reaction reagent was pyridylaminated using a Pyridylamination Reagent kit (Takara Bio, Japan) according to the manufacturer's protocol [50], [51]. The solutions for reductive amination were prepared as follows. 2-Aminopyridine (300 mg) was dissolved in 100 µL of acetic acid at 80°C and borane-dimethylamine complex (20 mg) was dissolved in 100 µL of acetic acid. Reductive amination was performed by the addition of 20 µL 2-aminopyridine solution to the treated fractions followed by heating at 90°C for 1 h, and the subsequent addition of 20 µL borane-dimethylamine complex solution with further heating of the mixtures at 80°C for 1 h. Following concentration, the mixtures were applied to a cellulose cartridge column.

## Supporting Information

Figure S1 Mass spectra of tryptic peptides separated by Zip Tipμ-C18 with 10% (a), 30% (b), 50% (c), and 70% CH_3_CN (d) after treatment with PNGase F of concentrated glycopeptides. The numbers indicate the peptide numbers in Table 1, where a, b, and c indicate the oxidized Met [M(Met)+1(H)+16(O)]^+^, [M(Met)+1(H)+16(O)-64(CH_3_SOH)]^+^, and the pyroglutamate [Q(Gln)+1(H)-17(NH_3_)]^+^, respectively.
(0.14 MB TIF)

Figure S2 Tandem mass spectrum of the signal (*m/z* 1341.5) appeared after AspN treatment that was the peptide sequence containing N^339^ from a peptide Y^334^–K^364^ (No. 4 in Table 1).
(0.10 MB TIF)

Figure S3 Tandem mass spectra of the glycopeptides of *m/z* 2858.0, which consisted of GlcM9Gn2 attached to N^1171^ISLDIR (a) and that of *m/z* 3703.2, which consisted of GnM3FGn2 attached to TLDISHNVIWSLSGN^267^ETYEIK^g^ (b).
(0.23 MB TIF)

Figure S4 RP-HPLC chromatogram of a glycopeptide-enriched fraction of Chp from *CG10166* knock down mutant (22 µg) (a) and that from GFP-IR control (15 µg) (b). *: The peak containing glycopeptides.
(0.22 MB TIF)
