# Supplementary figures and images for: Insight into the Regulation of Glycan Synthesis in Drosophila Chaoptin Based on Mass Spectrometry
Source: PLoS One. 2009 May 5;4(5):e5434. doi: 10.1371/journal.pone.0005434 (PMC2672165; doi:10.1371/journal.pone.0005434)

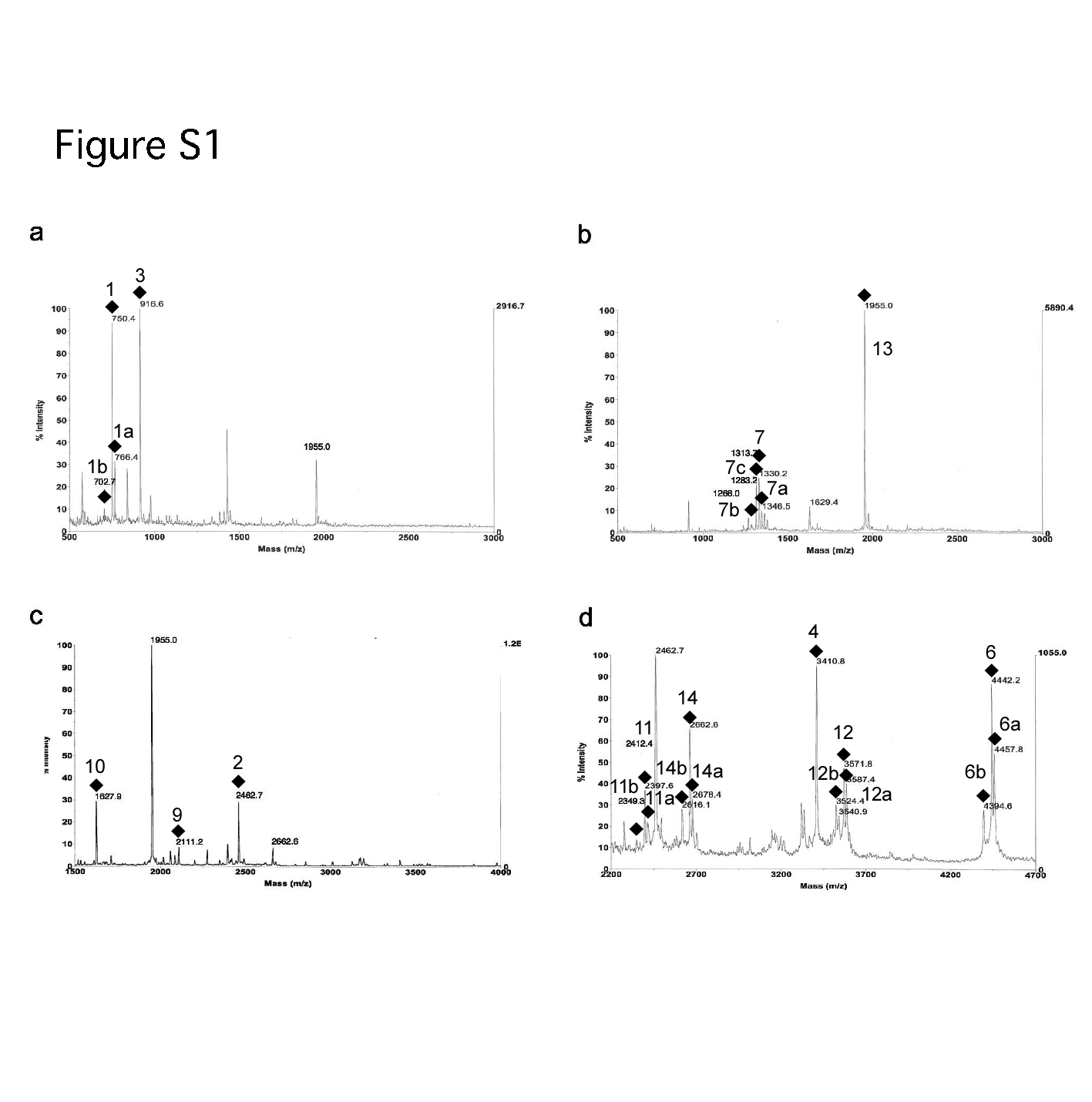

Supplement: Figure S1 — Mass spectra of tryptic peptides separated by Zip Tipμ-C18 with 10% (a), 30% (b), 50% (c), and 70% CH3CN (d) after treatment with PNGase F of concentrated glycopeptides. The numbers indicate the peptide numbers in Table 1, where a, b, and c indicate the oxidized Met [M(Met)+1(H)+16(O)]+, [M(Met)+1(H)+16(O)-64(CH3SOH)]+, and the pyroglutamate [Q(Gln)+1(H)-17(NH3)]+, respectively. (0.14 MB TIF) [file pone.0005434.s001.tif]

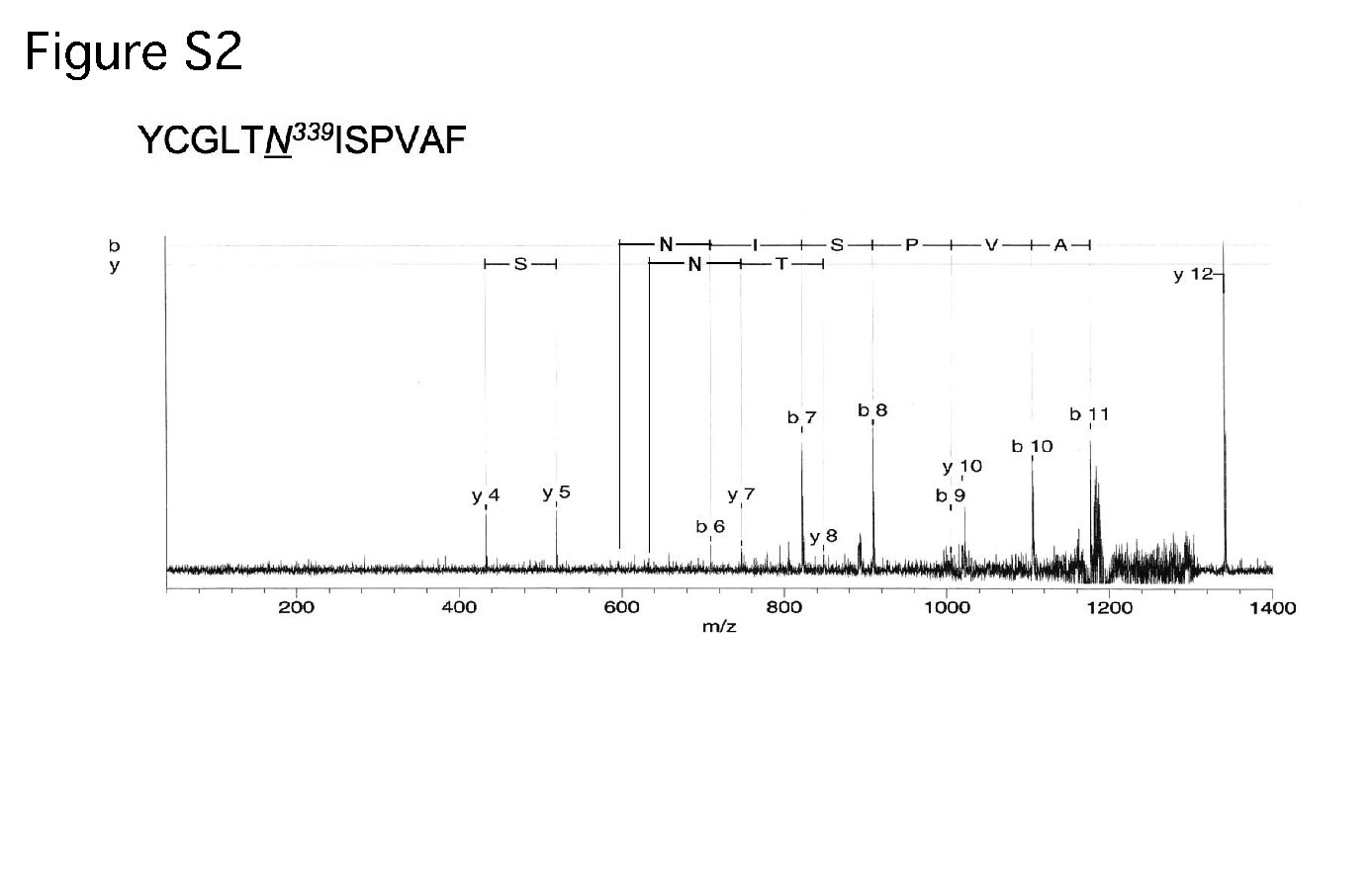

Supplement: Figure S2 — Tandem mass spectrum of the signal (m/z 1341.5) appeared after AspN treatment that was the peptide sequence containing N339 from a peptide Y334–K364 (No. 4 in Table 1). (0.10 MB TIF) [file pone.0005434.s002.tif]

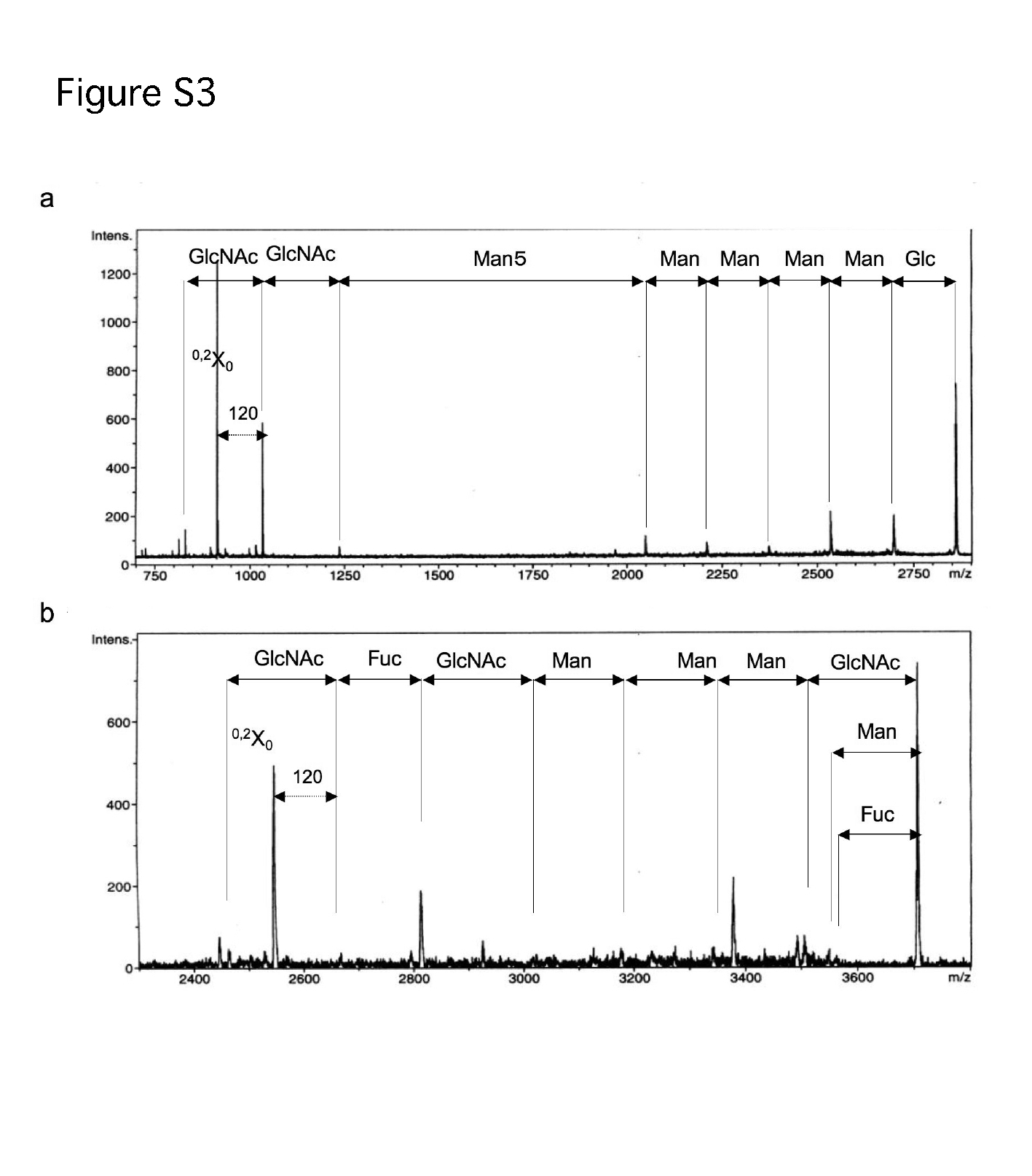

Supplement: Figure S3 — Tandem mass spectra of the glycopeptides of m/z 2858.0, which consisted of GlcM9Gn2 attached to N1171ISLDIR (a) and that of m/z 3703.2, which consisted of GnM3FGn2 attached to TLDISHNVIWSLSGN267ETYEIKg (b). (0.23 MB TIF) [file pone.0005434.s003.tif]

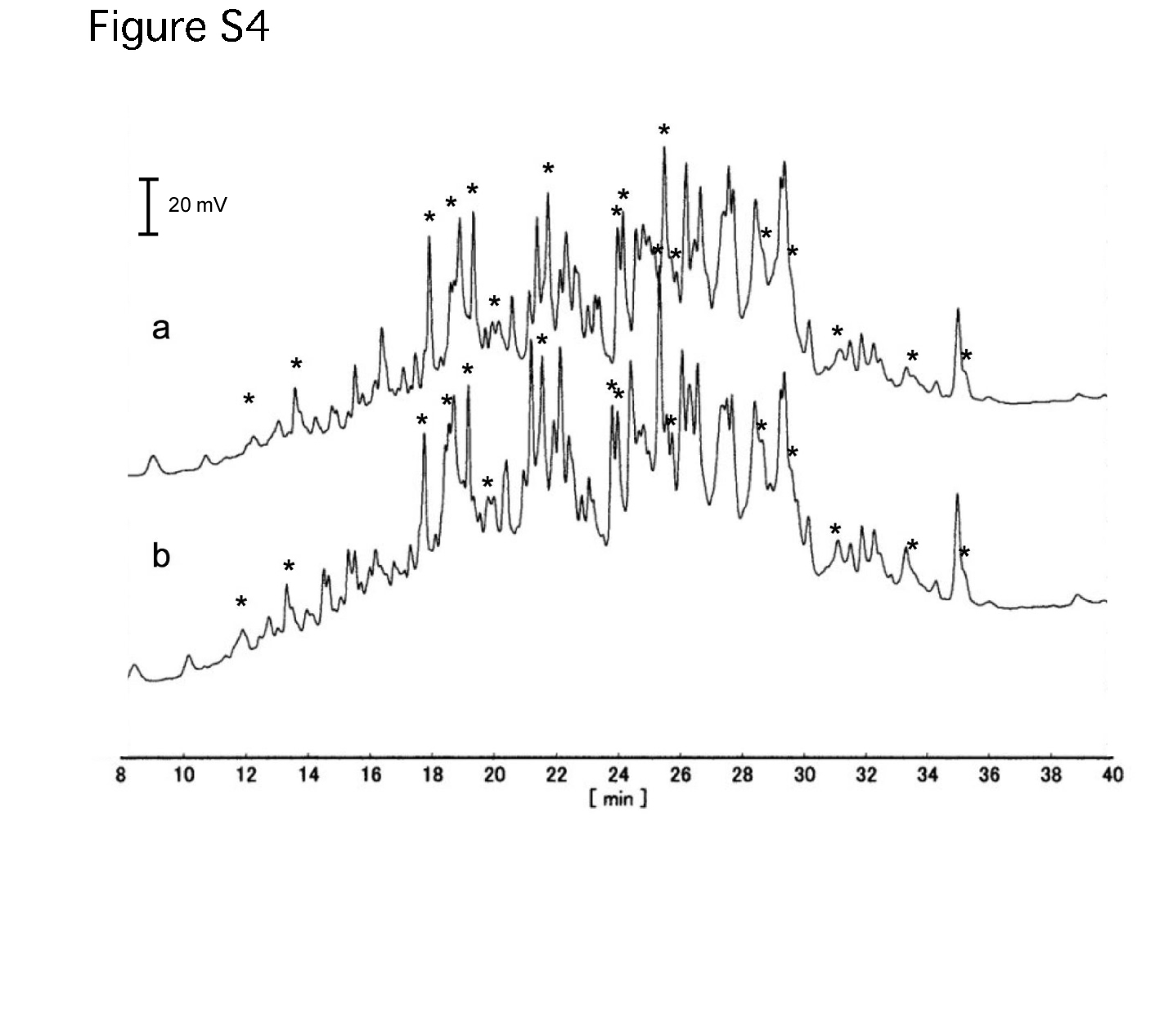

Supplement: Figure S4 — RP-HPLC chromatogram of a glycopeptide-enriched fraction of Chp from CG10166 knock down mutant (22 µg) (a) and that from GFP-IR control (15 µg) (b). *: The peak containing glycopeptides. (0.22 MB TIF) [file pone.0005434.s004.tif]
